# Serum interleukin-6 levels are increased in post-herpetic neuralgia: a single-center retrospective study^☆^

**DOI:** 10.1016/j.abd.2022.03.007

**Published:** 2023-01-18

**Authors:** Ding Lin, Changyang Zhong, Quanlong Jiang, Aihua Huang, Yuan Liu

**Affiliations:** a Department of Cardiology, Hangzhou Third People’s Hospital, Hangzhou, China; b Department of Cerebrovascular disease, Hangzhou Third People’s Hospital, Hangzhou, China; c Hangzhou Binjiang District Changhe Street Community Health Service Center, Hangzhou, China

**Keywords:** Interleukin-6, Neuralgia, postherpetic, Herpes zoster

## Abstract

**Background:**

Studies have shown that the overall incidence rate of herpeszoster (HZ) in China is 6.64 cases per 1000 people, despite such harms brought by postherpetic neuralgia (PHN), the mechanism of the disease remains unclear in China. Currently, effective biomarkers to predict PHN remain unavailable, which makes it difficult to prevent and successfully treat PHN.

**Objective:**

The aim of the study was to determine the serum interleukin-6 level in PHN.

**Methods:**

The serum levels of interleukin 6 (IL-6) were measured by multi-antibody sandwich ELISA. The likert scale was used to represent the degree of neuralgia in the patients. Patients with PHN were divided into a mild PHN group and a severe PHN group according to the Likert scale. ROC curve was performed for evaluating the diagnostic efficiency of IL6 for PHN. The correlation between the IL6 level and the Likert scale before and after treatment with gabapentin and mecobalamin was analyzed.

**Results:**

IL6 levels in PHN patients resulted higher compared to volunteers. Patients in the severe PHN group had a higher serum IL6 level than in the mild PHN group. The Likert scale score was related to the serum IL6 levels and the frequency of IL6 levels above the cutoff value (4.95 pg/mL) in PNH groups before and after treatment (p < 0.05).

**Study limitations:**

Pain is subjective. Some mental states, such as anxiety and depression, greatly influence an individual’s perception of pain, and pain tolerance can vary between people. Therefore, pain scores can be affected by different individual factors.

**Conclusions:**

The serum IL6 levels may be used as a biochemical indicator of the severity of PNH.

## Introduction

VZV is a kind of human alpha herpes virus. When a person is infected with VZV for the first time, which usually happens in childhood, varicella occurs. Herpeszoster (HZ) is caused by the reactivation of latent VZV in the cranial nerve or dorsal root ganglia in adulthood,^1^ possibly leading to Postherpetic Neuralgia (PHN). Even after the local skin lesions are repaired, the local skin neuralgia still persists for months or years or even develops into refractory neuralgia that often occurs in middle-aged and elderly people and causes long-lasting pain, especially in elderly women, besides, refractory neuralgia also leads to anorexia, insomnia, and depression, which cast a serious impact on the patient's life. Recent studies have shown that PHN is a kind of neuropathic pain^2^ and the risk factors of PHN include age, gender, rash precursor symptoms, severe skin damage, severe acute pain, and psychosocial factors.^3^ The incidence rate of PHN is about 2‒4.6 cases per thousand people per year, and that rate increases greatly to 9.1 cases per thousand people per year in the age group of 50 to 75 years old.^4^ Studies have shown that the overall incidence rate of PHN in China is 6.64 cases per 1000 people. Besides, there were 99 recurrent episodes in 4313 first episodes from 2015 to 2017. However, the average number of visits of patients exhibiting initial disease onset was significantly lower than the recurrent patients (3.6 vs. 6.7 per patient), and the average hospitalization time for recurrent attacks is longer than the first attack. About 9%‒34% of patients will develop PHN, and the rates of incidence and prevalence increase gradually with age.^5^

Despite such harms brought by PHN, the mechanism of the disease remains unclear in China. Currently, effective biomarkers to predict PHN remain unavailable, which makes it difficult for PHN prevention and successful PHN treatment. By observing the changes in serum cytokine levels in patients with postherpetic neuralgia, the relationship between cytokine levels and the pain degrees in PHN patients was investigated to find out the immunological factors affecting the onset of PHN, thereby establishing the foundation for early clinical intervention.

## Methods

### Patients and eligibility criteria

All participants provided oral and written informed consent before participating in the study. The design of this study conformed to the Declaration of Helsinki, and the study was approved by the Biomedical Ethics Committee of Hangzhou Third People's Hospital 80 patients (35 males and 45 females) with postherpetic neuralgia in the hospital from April 2019 to October 2021 were taken as the subjects in this study. The patients’ ages ranged from 40 to 85 years old with an average age of (68.3 ± 10.1) years old.

Inclusion criteria for PHN patients: 1) According to the diagnostic criteria of PHN, PHN in this study refers to pain at the rash site one month after HZ has been cured. 2) The patients must show no history of organic diseases, such as heart, lung, or kidney diseases. 3) The patients must show no obstacle in cognitive function and speech according to their Likert scale scores. Besides, they must be able to understand and accept their Likert scale scores. 4) The patients must not suffer from any other immune system diseases, cancers, bacterial infections, or other relevant symptoms.

Exclusion criteria for PHN patients: 1) Patients under 40 or over 80 years old. 2) Patients with cognitive and language dysfunction or unable to give Likert scale scores. 3) Cancer patients or patients with autoimmune diseases. 4) Recent use of immunosuppressants. 5) Pregnant and lactating women.

The likert scale is often used in clinical studies and case reports of postherpetic neuralgia. The score range of this scale is 0 (completely painless) to 100 (the most severe pain that may occur); Clinically, the minimum threshold of meaningful postherpetic neuralgia is 40 points, Clinically meaningful pain means that the Likert scale score is greater than 40 points; Severe pain means that the Likert scale score is greater than 70 points.^6^

Patients with PHN were divided into two groups according to the Likert scale. Patients with a Likert scale score of more than 70 points were defined as having severe PHN. Others with a Likert scale of more than 40 points were defined as mild PHN (n = 40), The PHN group was tested by a Likert scale on the day of hospitalization.

### Detection of serum IL6 levels and biochemical indicators

For each patient, 3 mL of venous blood was collected on the second day of admission before treatment. Another 3 mL of venous blood was obtained from the patient’s 14^th^ day after treatment, and all the blood samples were put into anticoagulant tubes and sent to the hospital specimen bank for separation and cryopreservation. The serum levels of IL-6 were measured by multi-antibody sandwich ELISA. The human MCP-1/IP-10/rnaates/fractalkine ELISA kit used in this study was purchased from Genzyme, USA, and the kit included distilled water, a sampler, an oscillator, and a magnetic stirrer. Biochemical indicators were detected by an automatic biochemical analyzer in the clinical laboratory of the hospital, including Triglyceride (TG), Total Cholesterol (TC), Tumor Necrosis Factor-α (TNF-α), Low-Density Lipoprotein Cholesterol (LDL-C), Interleukin-10 (IL10). The conditions of patients after treatment were assessed by a Likert scale score. The correlation between serum IL6 levels at admission and the Likert scale score after treatment was also analyzed.

All patients were administered appropriate doses of oral gabapentin and 1000 ug of intramuscular injection of mecobalamin daily. Patients with severe pain were additionally given appropriate doses of oral sustained-release tablets of oxycodone hydrochloride.

### Clinical data collection

Detailed medical history, medication history, and physical examination were performed on all patients and healthy volunteers. Clinical data included age, gender, smoking history, drinking history, and blood pressure.

### Statistical analysis

Statistical analyses were performed using SPSS version 21.0 (International Business Machines, corp., Armonk, NY, USA). Patient characteristics were described as frequencies (percentages) or mean ± SD. Significant differences between groups were assessed by One-way analysis of variance (ANOVA). The Chi-Square test was used for counting data. The diagnostic efficiency was calculated by Receiver Operating Characteristic Curve (ROC); a p-value <0.05 was considered statistically significant. Figures were drawn by SPSS Statistics and GraphPad Prism 8.0.

## Results

A total of 80 patients with PHN and 80 healthy volunteers were enrolled in this study. The characteristics of patients and volunteers were shown in Table 1.

**Table 1:** The detailed clinical information of patients in postherpetic neuralgia and healthy volunteers. (Mean ± SD).

Characters	Mild postherpetic neuralgia (n = 40)	Severe postherpetic neuralgia (n = 40)	Healthy volunteers (n = 80)
Gender			
Male	17	18	36
Female	23	22	44
Age	68.3 ± 10.1	70.3 ± 8.4	69.5 ± 9.2
Likert scale score	44.5 ± 12.3	78.2 ± 7.3	/
Drinking	12	14	20
Smoking	26	25	44
Hypertension	24	18	/
Diabetes	17	20	/
TC (mmoL/L)	4.52 ± 1.21	4.69 ± 0.69	4.01 ± 1.20
IL10 (pg/mL)	4.03 ± 1.14	4.12 ± 1.22	4.01 ± 1.14
TG (mmoL/L)	2.21 ± 1.26	2.47 ± 1.04	1.58 ± 0.97
TNF-α (pg/mL)	7.11 ± 1.11	7.41 ± 1.24	7.03 ± 1.12
LDL-C (mmoL/L)	3.01 ± 1.18	3.95 ± 1.09	2.82 ± 1.07

TG, Triglyceride; TC, Total Cholesterol; LDL-C, Low-Density Lipoprotein Cholesterol; IL10, Interleukin-10; TNF-α, Tumor Necrosis Factor-α.

There were no marked differences between patients with PHN and volunteers in variables, including age, gender, TNF-α, TC, TG, IL10 and LDL-C (p > 0.05, respectively).

**Figure 1:**
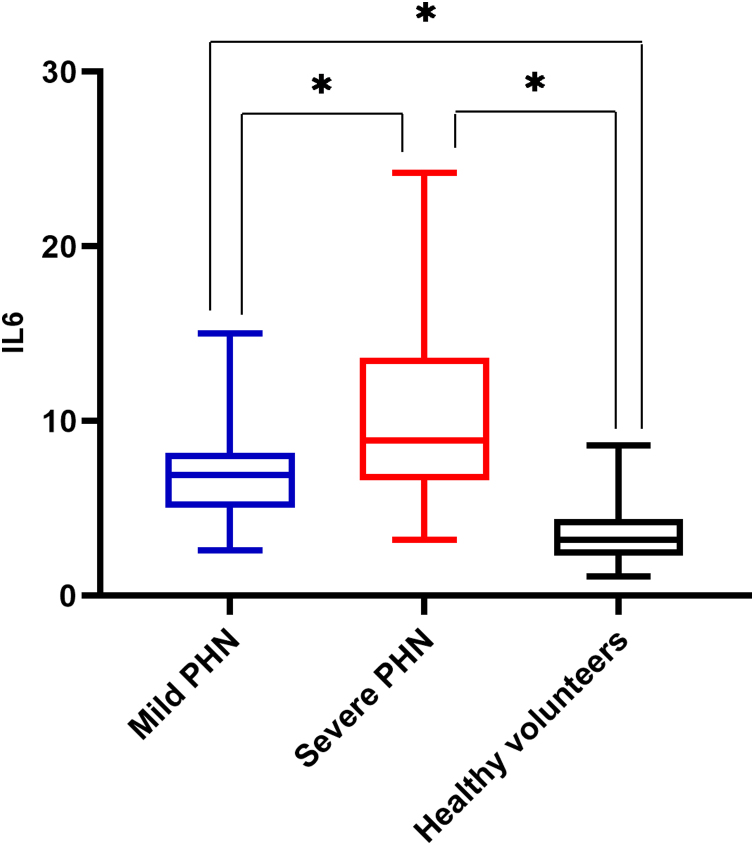
Serum IL6 levels in mild PHN, severe PHN and healthy volunteers’ groups (*p < 0.05).

**Figure 2:**
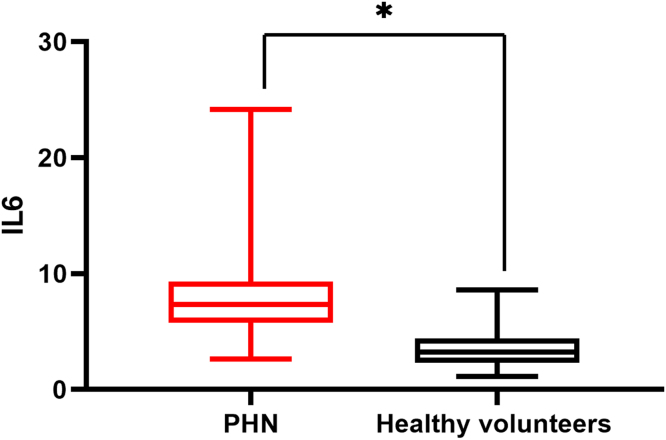
Serum IL6 levels in Postherpetic Neuralgia (PHN) and healthy volunteers’ groups (*p < 0.05).

There was a significant difference in serum IL6 levels between mild PHN and severe PHN groups, and patients in the severe PHN group had higher serum IL6 levels than those in the mild PHN group (10.41 ± 4.97 vs. 6.65 ± 2.25, p < 0.05). And patients with severe or mild PHN had higher serum IL6 levels than healthy volunteers p < 0.05 (Fig. 1), Serum IL6 levels in patients with PHN were higher than those in volunteers (7.35 ± 2.15 vs. 3.27 ± 1.02, p = 0.00) (Fig. 2).

**Figure 3:**
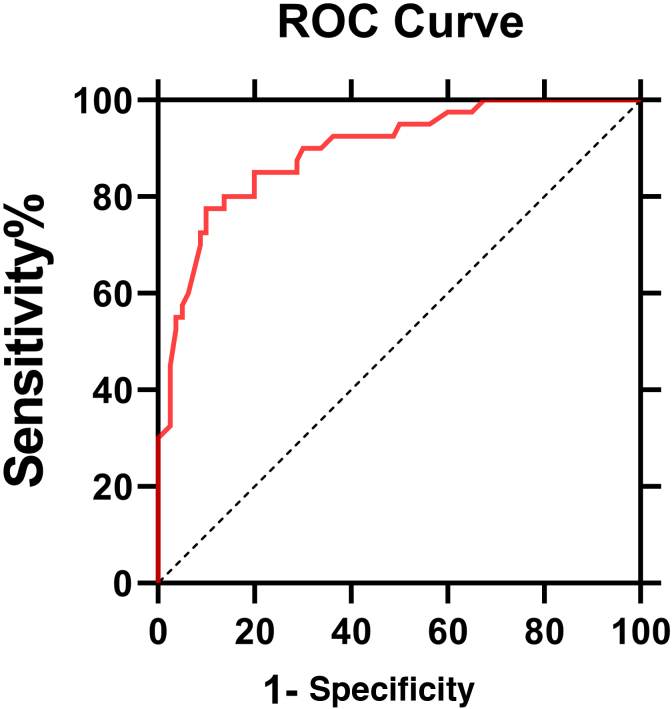
Receiver operator curve of IL6 for postherpetic neuralgia. The area under the curve values was 0.896 (95% CI 0.85‒0.95, p < 0.0001).

The ROC curve of IL6 levels for PHN was shown in Fig. 3. The AUC value was 0.896 (95% Confidence Interval: 0.85‒0.95, p < 0.0001). According to the ROC curve, the cut-off value of serum IL6 levels for PHN was 4.95 pg/mL, with 80% sensitivity and 86.2% specificity.

In the PHN group, an appropriate dose of gabapentin was given orally based on the patient's conditions, and Mecobalamin was given via intramuscular injections at a daily dose of one. After the 14^th^ treatment, all patients with PHN were assessed by a Likert scale score. The results were shown in Table 2. Serum IL6 levels at admission were associated with a Likert scale score and on the 14^th^ day after treatment (p < 0.05).

**Table 2:** Before and after 14 days of treatment, serum interleukin-6 levels in each group were correlated with Likert Scale score.

Likert scale score (point)	0‒40	40‒70	≥70	p
**On admission**				
Patient (case)	80	40	40	
Serum IL6 level (pg/mL)	3.27 ± 1.02	6.65 ± 2.25	10.41 ± 4.97	< 0.05
Serum IL6 ≥ 4.95 pg/mL (case)	4 (5%)	29 (72.5%)	37(92.5%)	
**14 days after treatment**				
Patient (case)	98	47	15	
Serum IL6 level (pg/mL)	3.18 ± 1.13	4.32 ± 1.72	7.01 ± 2.02	< 0.05
Serum IL6 ≥ 4.95 pg/mL (case)	0	10 (21.3%)	14 (93.3%)	

Patients with a Likert scale score of more than 70 points were defined as having severe PHN.

Others with a Likert scale of more than 40 points were defined as mild PHN.

The cut-off value of serum IL6 levels for PHN was 4.95 pg/mL.

## Discussion

Postherpetic neuralgia is a kind of refractory neuropathic pain. In recent years, studies have shown that PHN could be associated with several nervous system damage.^7^ PHN can have a variety of systemic symptoms, including chronic fatigue, anorexia, weight loss, lack of activity, and insomnia, accompanied by emotional or psychological changes, such as anxiety, depression, inattention, etc. some patients have suicidal tendencies.^8^ In China, it has been reported that about 45% of patients suffered moderate to severe emotional interference, 40% of patients with postherpetic neuralgia suffered moderate to severe interference in their daily living activities, more than 40% of patients were accompanied by moderate to severe sleep disorders and 59.68% of patients had or often had suicidal thoughts.^9^ It is also reported in foreign literature the more severe the pain, the more serious the impact on patients' energy, sleep, and overall quality of life. In addition, it can have a moderate to severe impact on family members' lives, causing them to experience fatigue, stress, insomnia, and emotional distress.^10^

A variety of inflammatory substances has been revealed to involve neuropathic pain.^11^ Specifically, cytokines are capable of regulating a variety of cellular physiological functions. They are polypeptides with small molecular weights that are synthesized and secreted by all kinds of cells in the body, playing an important role in the stress processes of trauma, pain, and infection.^12^ At present, numerous types of cytokines have been discovered, including Interleukin (IL), Interferon (IFN), Colony Stimulating Factor (CSF), Tumor Necrosis Factor (TNF), Nerve Growth Factor (NGF), and Transforming Growth Factor (TGF). They are found to engage in the regulation of homeostasis in the immune system, and if excessive stress occurs, pathological reactions will happen. Earlier studies have shown that cytokines can be categorized as pro-inflammatory or anti-inflammatory.^13^ The former includes IL-6, IL-1, IL-8, and TNF, while the latter involves IL-4, IL-10, soluble Interleukin-2 receptor antagonist (IL-2), and tumor necrosis factor. Under normal physiological conditions, these two types of cytokines keep a fine balance with the help of neuroendocrine and humoral regulations.^14^ It is noteworthy that IL-6 is a multi-effector cytokine produced by Th2 cells, monocyte macrophages, and endothelial cells. It is a crucial molecule in immune response, acute phase response, and hematopoietic regulation. Besides, as a differentiation and growth factor of B-cells, T-cells, and endothelial cells, IL-6 can activate target genes, participate in humoral immunity, induce megakaryocyte maturation, and act on the growth, differentiation, regeneration, and degradation of nerve cells in both peripheral and central nervous systems. Under normal physiological conditions, the IL-6 content secreted by macrophages, mast cells, lymphocytes, neurons, and glial cells is at a low level, which facilitates the normal development and repair of the nervous system, while a high level of IL-6 content can cause damage to the nervous system. Researchers also found that the level of IL-6 in PHN or posterior neuralgia patients was positively correlated with sensory loss and loss of cold sensation in the area of pain.^15^, ^16^, ^17^, ^18^, ^19^

Under peripheral nerve injury, the expression of IL-6 and IL-6 receptors in the spinal cord is up-regulated. In animal experiments, IL-6 injections into normal rats can inhibit their responses to thermal and mechanical stimulation. Even when the pain responses caused by mechanical stimulation were preserved, IL-6 injections could still inhibit the pain responses caused by thermal stimulation. Therefore, IL-6 has been verified to play an important role in the persistent pain resulting from peripheral and central nerve injury.^20^, ^21^ In addition Arruda et al.^22^ conducted several animal experiments, in which IL-6 antibodies or allogeneic IgG were applied to treat rats with persistent hyperalgesia due to peripheral nerve injury.

In the present study, it was found that the serum IL-6 level was positively correlated with the Likert scale score, suggesting the role of IL-6 in the pathological progression of PHN. Some cytokines such as IFN-gamma, IL-6, and IL-8 were slightly raised in the zoster group compared with a group of normal healthy subjects of similar age distribution, these differences only verged on significance.^23^ It was partially consistent with the present study, but this study further confirmed that IL6 was closely related to PHN through stratified grouping. In the present study, the level of serum IL-6 in patients with severe PHN was significantly higher than that in patients with mild PHN or the healthy control group, and they were higher in patients with severe PHN than those in patients with mild PHN. Suggesting that PHN patients with high IL-6 levels were more likely to develop PHN.

In the present study, the serum IL-6 level showed a statistically significant difference between PHN patients before and after treatment. Saxena et al. studied the treatment process of herpes zoster neuralgia and used Cognitive Behavioral Therapy (CBT) along with pregabalin. The pain relief effect is remarkable, a significant downregulation of mRNA expression of IL-6 was observed.^24^ Confirming consistent results in East Asian and West Asian populations, however, it is not shown that IL6 level is closely related to PHN prognosis. This study found that serum IL6 levels in patients with PHN in admission are correlated to the Likert scale score on the 14^th^ day after diagnosis. It is indicated that serum IL6 levels in patients with PHN in admission may be a predictive factor for short-term prognosis.

Serum IL6 may be a diagnostic indicator of PHN with 85% sensitivity and 87.5% specificity. The possibility of herpes zoster developing into PHN can be judged early by the level of IL6. Early intervention and regular treatment of patients with elevated IL-6 levels could help alleviate their pain and improve their quality of life.

## Conclusions

To sum up, the level of serum interleukin-6 is related to the degree of postherpetic neuralgia and may be a useful diagnostic biomarker in such cases.

## Financial support

Zhejiang Provincial Health Science and Technology Project 2022KY977.

## Authors’ contributions

Ding Lin: Data collection, analysis, and interpretation.

Changyang Zhong: Approval of the final version of the manuscript; critical literature review; manuscript critical review.

Quanlong Jiang: Effective participation in research orientation.

Aihua Huang: Intellectual participation in propaedeutic and/or therapeutic management of studied cases.

Yuan Liu: Preparation and writing of the manuscript; statistical analysis.

Yan Zhang: Study conception and planning.

## Conflicts of interest

None declared.
